# 2-(4-Chloro­benzoyl­meth­yl)-2*H*-1,4-benzothia­zin-3(4*H*)-one

**DOI:** 10.1107/S1600536808007423

**Published:** 2008-03-29

**Authors:** Ping Zhang, Na Du, Lan-Zhi Wang, Yuan Li

**Affiliations:** a College of Chemistry and Materials Science, Hebei Normal University, Shijiazhuang 050016, People’s Republic of China; b Department of Chemical Engineering, Shijiazhuang Vocational Technology Institute, Shijiazhuang 050081, People’s Republic of China

## Abstract

The six-membered heterocyclic ring in the title compound, C_16_H_12_ClNO_2_S, exists in a conformation intermediate between twist-boat and chair. A one-dimensional chain structure is formed as a result of inter­molecular N—H⋯O and C—H⋯O hydrogen bonds *via* crystallographic inversion symmetry and translation along the *a* axis.

## Related literature

For the synthesis and biological activities of related chalcones and 1,5-benzothia­zepines, see: Ansari *et al.* (2005 ▶); Pant *et al.* (2006 ▶). For microwave-assisted syntheses of related compounds, see: Dandia *et al.* (2002 ▶). For further related literature, see: Pant & Chugh (1989 ▶); Kirchner & Alexander (1959 ▶); Beryozkina *et al.* (2004 ▶); Pant *et al.* (1987 ▶).
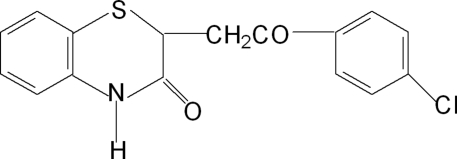


## Experimental

### 

#### Crystal data

C_16_H_12_ClNO_2_S
*M* *_r_* = 317.78
Triclinic, 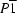

*a* = 7.7273 (19) Å
*b* = 8.649 (2) Å
*c* = 12.298 (3) Å
α = 82.032 (3)°
β = 72.349 (2)°
γ = 68.829 (3)°
*V* = 730.0 (3) Å^3^
*Z* = 2
Mo *K*α radiation
μ = 0.41 mm^−1^
*T* = 273 (2) K
0.24 × 0.20 × 0.18 mm

#### Data collection

Bruker APEXII CCD area-detector diffractometer
Absorption correction: multi-scan (*SADABS*; Sheldrick, 1997 ▶) *T*_min_ = 0.876, *T*_max_ = 1.000 (expected range = 0.814–0.929)
3962 measured reflections
2545 independent reflections
2236 reflections with *I* > 2σ(*I*)
*R*_int_ = 0.009

#### Refinement

*R*[*F* ^2^ > 2σ(*F* ^2^)] = 0.030
*wR*(*F* ^2^) = 0.082
*S* = 1.06
2545 reflections
190 parameters
H-atom parameters constrained
Δρ_max_ = 0.18 e Å^−3^
Δρ_min_ = −0.27 e Å^−3^

### 

Data collection: *APEX2* (Bruker, 1997 ▶); cell refinement: *SAINT* (Bruker, 1997 ▶); data reduction: *SAINT*; program(s) used to solve structure: *SHELXS97* (Sheldrick, 2008 ▶); program(s) used to refine structure: *SHELXL97* (Sheldrick, 2008 ▶); molecular graphics: *SHELXTL* (Sheldrick, 2008 ▶); software used to prepare material for publication: *SHELXTL* and *PLATON* (Spek, 2003 ▶).

## Supplementary Material

Crystal structure: contains datablocks global, I. DOI: 10.1107/S1600536808007423/si2076sup1.cif

Structure factors: contains datablocks I. DOI: 10.1107/S1600536808007423/si2076Isup2.hkl

Additional supplementary materials:  crystallographic information; 3D view; checkCIF report

## Figures and Tables

**Table 1:** Hydrogen-bond geometry (Å, °)

*D*—H⋯*A*	*D*—H	H⋯*A*	*D*⋯*A*	*D*—H⋯*A*
N1—H1⋯O1^i^	0.86	2.02	2.8812 (19)	177
C7—H7⋯O2^ii^	0.98	2.54	3.443 (2)	152
C9—H9*A*⋯O1^iii^	0.97	2.59	3.485 (2)	153
C13—H13⋯O1^iv^	0.93	2.59	3.400 (2)	145

Symmetry codes: (i) 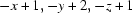; (ii) 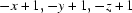; (iii) 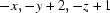; (iv) 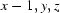.

**Fig. 1.**
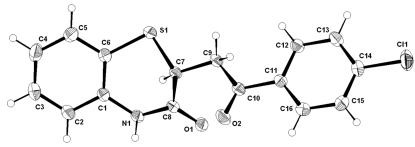
Molecular structure of the title compound with displacement ellipsoids drawn at the 50% probability level.

## supplementary crystallographic information

### Comment

1,5-Benzothiazepine and its derivatives are an important class of bioactive molecules in the field of drugs and pharmaceuticals (Ansari *et al.*, 2005; Pant *et al.*, 2006). The reaction of 2-aminothiophenol with various α,β-unsaturated carbonyl compounds lead to the formation of 1,5-benzothiazepines. We have synthesized some 3-acetyl and 3-alkoxycarbonyl substituted 1,5-benzothiazepines, showing very good activity against fungus candida albicans by the reaction of 2-aminothiophenol with acetylacetone and ethyl acetoacetate. In continuation of our ongoing studies on the synthesis of 4-aryl-2-carboxy-2,3-dihydro-1,5-benzothiazepines for various biological activities, we reacted 2-aminothiophenol with β-aroylacrylic acids, but the formation of a seven-membered ring did not occur. Several authors have reported about the 4-aryl-2-carboxy-2,3-dihydro-1,5-benzothiazepine structure for the products of the reaction of 2-aminothiophenol with β-aroylacrylic acids (Pant & Chugh, 1989; Dandia *et al.*, 2002), and others proposed the formation of 1,4-benzothiazin-3-ones (Kirchner & Alexander, 1959; Beryozkina *et al.*, 2004).

So we repeated the experiment for several times and recrystallized the final product again. Upon X-ray diffraction analysis, along with the spectroscopic data we know that 1,4-benzothiazin-3-one was obtained as the only product (Fig. 1). In the crystalline state, the title compound form a one-dimensional chain structure due to intermolecular N—H···O and C—H···O hydrogen bonds *via* crystallographic inversion symmetry and translation along the *a* axis (Table 1). The substituent at atom C7 is equatorially oriented with a torsion angle O1–C8–C7–C9 = 8.3 (2)°. The carbonyl group is slightly turned relative to the chlorophenyl substituent with a torsion angle O2–C10–C11–C16 = 10.8 (2)°. The six-membered heterocycle in the title compound exists in intermediate conformation between twisted boat and chair type.

### Experimental

4-(4-chlorophenyl)-4-oxo-2-butenoic acid was prepared by AlCl_3_ catalysed treatment of powdered meleic anhydride with chlorobenzene following the general literature procedure. (Pant *et al.*, 1987). 2-Aminothiophenol (2 mmol) and 4-(4-chlorophenyl)-4-oxo-2-butenoic acid (2 mmol) were refluxed with dry ethanol saturated with hydrogen chloride gas. Excess of solvent was concentrated by distillation under reduced pressure and the residue crystallized from methanol to give the title compound as light yellow crystals suitable for X-ray structure determination. Analysis calculated for C_16_H_12_ClNO_2_S: C 60.47, H 3.81, N 4.41%; found: C 60.45, H 3.80, N 4.40%.

### Refinement

All H atoms were placed in calculated positions and constrained to ride on their parent atoms, with C—H distances of 0.93 Å (aryl C) and 0.97–0.98 Å (C*sp*^3^), and N—H distance of 0.86 Å, with all *U*_iso_(H) = 1.2*U*_eq_(C*sp*^2^).

### Crystal data

**Table:**

C_16_H_12_ClNO_2_S	*Z* = 2
*M**_r_* = 317.78	*F*_000_ = 328
Triclinic, *P*1	*D*_x_ = 1.446 Mg m^−^^3^
*a* = 7.7273 (19) Å	Mo *K*α radiation λ = 0.71073 Å
*b* = 8.649 (2) Å	Cell parameters from 2700 reflections
*c* = 12.298 (3) Å	θ = 2.5–28.0º
α = 82.032 (3)º	µ = 0.41 mm^−^^1^
β = 72.349 (2)º	*T* = 273 (2) K
γ = 68.829 (3)º	Labellar, colorless
*V* = 730.0 (3) Å^3^	0.24 × 0.20 × 0.18 mm

### Data collection

**Table:**

Bruker APEXII CCD area-detector diffractometer	2545 independent reflections
Radiation source: fine-focus sealed tube	2236 reflections with *I* > 2σ(*I*)
Monochromator: graphite	*R*_int_ = 0.009
*T* = 273(2) K	θ_max_ = 25.0º
φ and ω scans	θ_min_ = 1.7º
Absorption correction: multi-scan(SADABS; Sheldrick, 1997)	*h* = −9→9
*T*_min_ = 0.876, *T*_max_ = 1.000	*k* = −10→8
3962 measured reflections	*l* = −14→14

### Refinement

**Table:**

Refinement on *F*^2^	Secondary atom site location: difference Fourier map
Least-squares matrix: full	Hydrogen site location: inferred from neighbouring sites
*R*[*F*^2^ > 2σ(*F*^2^)] = 0.030	H-atom parameters constrained
*wR*(*F*^2^) = 0.082	*w* = 1/[σ^2^(*F*_o_^2^) + (0.0412*P*)^2^ + 0.2045*P*] where *P* = (*F*_o_^2^ + 2*F*_c_^2^)/3
*S* = 1.06	(Δ/σ)_max_ < 0.001
2545 reflections	Δρ_max_ = 0.18 e Å^−^^3^
190 parameters	Δρ_min_ = −0.27 e Å^−^^3^
Primary atom site location: structure-invariant direct methods	Extinction correction: none

### Special details

**Table:**

Geometry. All e.s.d.'s (except the e.s.d. in the dihedral angle between two l.s. planes) are estimated using the full covariance matrix. The cell e.s.d.'s are taken into account individually in the estimation of e.s.d.'s in distances, angles and torsion angles; correlations between e.s.d.'s in cell parameters are only used when they are defined by crystal symmetry. An approximate (isotropic) treatment of cell e.s.d.'s is used for estimating e.s.d.'s involving l.s. planes.
Refinement. Refinement of *F*^2^ against ALL reflections. The weighted *R*-factor *wR* and goodness of fit *S* are based on *F*^2^, conventional *R*-factors *R* are based on *F*, with *F* set to zero for negative *F*^2^. The threshold expression of *F*^2^ > σ(*F*^2^) is used only for calculating *R*-factors(gt) *etc*. and is not relevant to the choice of reflections for refinement. *R*-factors based on *F*^2^ are statistically about twice as large as those based on *F*, and *R*- factors based on ALL data will be even larger.

### Fractional atomic coordinates and isotropic or equivalent isotropic displacement parameters (Å^2^)

**Table:**

	*x*	*y*	*z*	*U*_iso_*/*U*_eq_	
Cl1	−0.60724 (7)	0.61069 (8)	0.87177 (5)	0.0776 (2)	
S1	0.31357 (6)	0.84225 (6)	0.23145 (3)	0.04914 (15)	
O1	0.30018 (15)	0.92709 (13)	0.52142 (9)	0.0402 (3)	
O2	0.28795 (16)	0.57700 (15)	0.57928 (11)	0.0527 (3)	
N1	0.54966 (17)	0.90262 (15)	0.36402 (11)	0.0373 (3)	
H1	0.5980	0.9498	0.3985	0.045*	
C1	0.6460 (2)	0.86774 (18)	0.24884 (13)	0.0363 (3)	
C2	0.8326 (2)	0.8708 (2)	0.20508 (15)	0.0474 (4)	
H2	0.8953	0.8892	0.2532	0.057*	
C3	0.9255 (3)	0.8467 (2)	0.09073 (16)	0.0567 (5)	
H3	1.0508	0.8488	0.0619	0.068*	
C4	0.8333 (3)	0.8197 (3)	0.01912 (16)	0.0632 (5)	
H4	0.8959	0.8043	−0.0582	0.076*	
C5	0.6482 (3)	0.8155 (3)	0.06182 (15)	0.0571 (5)	
H5	0.5869	0.7963	0.0132	0.069*	
C6	0.5527 (2)	0.8397 (2)	0.17663 (13)	0.0418 (4)	
C7	0.3290 (2)	0.75703 (18)	0.37328 (12)	0.0344 (3)	
H7	0.4274	0.6463	0.3666	0.041*	
C8	0.3893 (2)	0.87005 (17)	0.42642 (12)	0.0327 (3)	
C9	0.1349 (2)	0.74330 (18)	0.44118 (12)	0.0353 (3)	
H9A	0.0417	0.8540	0.4564	0.042*	
H9B	0.0918	0.6900	0.3952	0.042*	
C10	0.1384 (2)	0.64667 (18)	0.55313 (13)	0.0361 (3)	
C11	−0.0490 (2)	0.63785 (17)	0.63063 (13)	0.0348 (3)	
C12	−0.2165 (2)	0.68862 (19)	0.59542 (14)	0.0399 (4)	
H12	−0.2133	0.7301	0.5212	0.048*	
C13	−0.3878 (2)	0.6784 (2)	0.66903 (15)	0.0448 (4)	
H13	−0.4992	0.7119	0.6448	0.054*	
C14	−0.3901 (2)	0.6180 (2)	0.77841 (15)	0.0457 (4)	
C15	−0.2254 (3)	0.5640 (2)	0.81540 (15)	0.0537 (4)	
H15	−0.2293	0.5219	0.8896	0.064*	
C16	−0.0555 (2)	0.5732 (2)	0.74112 (14)	0.0467 (4)	
H16	0.0563	0.5357	0.7652	0.056*	

### Atomic displacement parameters (Å^2^)

**Table:**

	*U*^11^	*U*^22^	*U*^33^	*U*^12^	*U*^13^	*U*^23^
Cl1	0.0481 (3)	0.1072 (5)	0.0628 (3)	−0.0307 (3)	0.0052 (2)	0.0103 (3)
S1	0.0396 (2)	0.0791 (3)	0.0395 (2)	−0.0307 (2)	−0.01448 (18)	0.0016 (2)
O1	0.0368 (6)	0.0468 (6)	0.0402 (6)	−0.0183 (5)	−0.0069 (5)	−0.0085 (5)
O2	0.0335 (6)	0.0611 (7)	0.0604 (8)	−0.0122 (5)	−0.0183 (5)	0.0097 (6)
N1	0.0339 (7)	0.0453 (7)	0.0402 (7)	−0.0202 (6)	−0.0103 (5)	−0.0062 (5)
C1	0.0336 (8)	0.0368 (8)	0.0399 (8)	−0.0150 (6)	−0.0090 (6)	0.0000 (6)
C2	0.0380 (9)	0.0573 (10)	0.0510 (10)	−0.0223 (8)	−0.0117 (7)	0.0011 (8)
C3	0.0409 (10)	0.0716 (12)	0.0548 (11)	−0.0273 (9)	−0.0001 (8)	0.0001 (9)
C4	0.0591 (12)	0.0848 (14)	0.0433 (10)	−0.0350 (11)	0.0042 (9)	−0.0065 (9)
C5	0.0593 (11)	0.0816 (13)	0.0404 (9)	−0.0376 (10)	−0.0095 (8)	−0.0051 (9)
C6	0.0378 (9)	0.0499 (9)	0.0403 (8)	−0.0205 (7)	−0.0080 (7)	−0.0003 (7)
C7	0.0299 (7)	0.0368 (8)	0.0389 (8)	−0.0132 (6)	−0.0095 (6)	−0.0036 (6)
C8	0.0284 (7)	0.0329 (7)	0.0381 (8)	−0.0099 (6)	−0.0121 (6)	0.0000 (6)
C9	0.0298 (8)	0.0371 (8)	0.0427 (8)	−0.0144 (6)	−0.0107 (6)	−0.0032 (6)
C10	0.0326 (8)	0.0345 (8)	0.0436 (8)	−0.0118 (6)	−0.0120 (6)	−0.0040 (6)
C11	0.0341 (8)	0.0318 (7)	0.0404 (8)	−0.0122 (6)	−0.0110 (6)	−0.0025 (6)
C12	0.0373 (8)	0.0420 (8)	0.0427 (8)	−0.0169 (7)	−0.0141 (7)	0.0078 (7)
C13	0.0346 (8)	0.0471 (9)	0.0542 (10)	−0.0166 (7)	−0.0147 (7)	0.0070 (7)
C14	0.0375 (9)	0.0491 (9)	0.0457 (9)	−0.0161 (7)	−0.0025 (7)	−0.0021 (7)
C15	0.0521 (11)	0.0704 (12)	0.0363 (9)	−0.0216 (9)	−0.0106 (8)	0.0047 (8)
C16	0.0418 (9)	0.0573 (10)	0.0431 (9)	−0.0155 (8)	−0.0170 (7)	0.0002 (7)

### Geometric parameters (Å, °)

**Table:**

Cl1—C14	1.7394 (17)	C7—C8	1.5135 (19)
S1—C6	1.7573 (16)	C7—C9	1.5173 (19)
S1—C7	1.8182 (15)	C7—H7	0.9800
O1—C8	1.2284 (18)	C9—C10	1.511 (2)
O2—C10	1.2129 (18)	C9—H9A	0.9700
N1—C8	1.3489 (18)	C9—H9B	0.9700
N1—C1	1.403 (2)	C10—C11	1.494 (2)
N1—H1	0.8600	C11—C16	1.389 (2)
C1—C2	1.386 (2)	C11—C12	1.390 (2)
C1—C6	1.394 (2)	C12—C13	1.384 (2)
C2—C3	1.377 (2)	C12—H12	0.9300
C2—H2	0.9300	C13—C14	1.371 (2)
C3—C4	1.377 (3)	C13—H13	0.9300
C3—H3	0.9300	C14—C15	1.381 (3)
C4—C5	1.378 (3)	C15—C16	1.376 (2)
C4—H4	0.9300	C15—H15	0.9300
C5—C6	1.386 (2)	C16—H16	0.9300
C5—H5	0.9300		
			
C6—S1—C7	97.48 (7)	O1—C8—C7	122.41 (13)
C8—N1—C1	126.91 (12)	N1—C8—C7	116.00 (12)
C8—N1—H1	116.5	C10—C9—C7	113.70 (12)
C1—N1—H1	116.5	C10—C9—H9A	108.8
C2—C1—C6	119.70 (14)	C7—C9—H9A	108.8
C2—C1—N1	119.38 (14)	C10—C9—H9B	108.8
C6—C1—N1	120.82 (13)	C7—C9—H9B	108.8
C3—C2—C1	120.31 (16)	H9A—C9—H9B	107.7
C3—C2—H2	119.8	O2—C10—C11	120.79 (14)
C1—C2—H2	119.8	O2—C10—C9	121.44 (14)
C4—C3—C2	120.09 (16)	C11—C10—C9	117.77 (12)
C4—C3—H3	120.0	C16—C11—C12	118.65 (14)
C2—C3—H3	120.0	C16—C11—C10	118.96 (13)
C3—C4—C5	120.09 (17)	C12—C11—C10	122.38 (14)
C3—C4—H4	120.0	C13—C12—C11	121.08 (15)
C5—C4—H4	120.0	C13—C12—H12	119.5
C4—C5—C6	120.49 (17)	C11—C12—H12	119.5
C4—C5—H5	119.8	C14—C13—C12	118.69 (15)
C6—C5—H5	119.8	C14—C13—H13	120.7
C5—C6—C1	119.31 (15)	C12—C13—H13	120.7
C5—C6—S1	121.07 (13)	C13—C14—C15	121.61 (15)
C1—C6—S1	119.58 (12)	C13—C14—Cl1	118.54 (13)
C8—C7—C9	112.96 (12)	C15—C14—Cl1	119.85 (14)
C8—C7—S1	107.61 (10)	C16—C15—C14	119.17 (16)
C9—C7—S1	108.66 (10)	C16—C15—H15	120.4
C8—C7—H7	109.2	C14—C15—H15	120.4
C9—C7—H7	109.2	C15—C16—C11	120.77 (15)
S1—C7—H7	109.2	C15—C16—H16	119.6
O1—C8—N1	121.58 (13)	C11—C16—H16	119.6
			
C8—N1—C1—C2	−164.11 (15)	C9—C7—C8—N1	−172.83 (12)
C8—N1—C1—C6	19.6 (2)	S1—C7—C8—N1	−52.90 (15)
C6—C1—C2—C3	0.2 (3)	C8—C7—C9—C10	−70.81 (16)
N1—C1—C2—C3	−176.10 (16)	S1—C7—C9—C10	169.86 (10)
C1—C2—C3—C4	0.1 (3)	C7—C9—C10—O2	−5.5 (2)
C2—C3—C4—C5	−0.5 (3)	C7—C9—C10—C11	175.14 (12)
C3—C4—C5—C6	0.5 (3)	O2—C10—C11—C16	10.8 (2)
C4—C5—C6—C1	−0.2 (3)	C9—C10—C11—C16	−169.83 (14)
C4—C5—C6—S1	177.63 (15)	O2—C10—C11—C12	−167.67 (15)
C2—C1—C6—C5	−0.1 (2)	C9—C10—C11—C12	11.7 (2)
N1—C1—C6—C5	176.12 (15)	C16—C11—C12—C13	1.3 (2)
C2—C1—C6—S1	−178.03 (12)	C10—C11—C12—C13	179.77 (14)
N1—C1—C6—S1	−1.8 (2)	C11—C12—C13—C14	0.4 (2)
C7—S1—C6—C5	148.48 (15)	C12—C13—C14—C15	−1.5 (3)
C7—S1—C6—C1	−33.66 (14)	C12—C13—C14—Cl1	178.29 (12)
C6—S1—C7—C8	58.07 (11)	C13—C14—C15—C16	0.9 (3)
C6—S1—C7—C9	−179.31 (10)	Cl1—C14—C15—C16	−178.92 (14)
C1—N1—C8—O1	−169.12 (14)	C14—C15—C16—C11	0.8 (3)
C1—N1—C8—C7	12.0 (2)	C12—C11—C16—C15	−1.9 (2)
C9—C7—C8—O1	8.3 (2)	C10—C11—C16—C15	179.53 (15)
S1—C7—C8—O1	128.25 (13)		

### Hydrogen-bond geometry (Å, °)

**Table:**

*D*—H···*A*	*D*—H	H···*A*	*D*···*A*	*D*—H···*A*
N1—H1···O1^i^	0.86	2.02	2.8812 (19)	177
C7—H7···O2^ii^	0.98	2.54	3.443 (2)	152
C9—H9A···O1^iii^	0.97	2.59	3.485 (2)	153
C13—H13···O1^iv^	0.93	2.59	3.400 (2)	145

Symmetry codes: (i) −*x*+1, −*y*+2, −*z*+1; (ii) −*x*+1, −*y*+1, −*z*+1; (iii) −*x*, −*y*+2, −*z*+1; (iv) *x*−1, *y*, *z*.
